# Postless Tape Augmentation for Anterior Cruciate Ligament (ACL) Reconstruction

**DOI:** 10.1016/j.eats.2022.08.021

**Published:** 2022-11-17

**Authors:** Rami G. Alrabaa, Ajay S. Padaki, Abhishek Kannan, Alan L. Zhang

**Affiliations:** Department of Orthopaedic Surgery, University of California–San Francisco, San Francisco, California, U.S.A.

## Abstract

Suture or tape augmentation for anterior cruciate ligament (ACL) reconstruction has been described as a technique to increase biomechanical strength and potentially improve clinical outcomes. However, the suture or tape used for augmentation usually requires independent tibial fixation from the ACL graft in the form of an anchor or post. This may introduce the potential for graft and augment tension mismatch, while increasing surgical cost. We present our technique for ACL reconstruction with postless tape augmentation. The ACL graft and tape are fixed at the same tension with interference fixation using a single tibial sheath and screw construct, which allows for ACL augmentation without the need for an additional post or implant.

## Introduction

Anterior cruciate ligament (ACL) ruptures are among the most frequent injuries seen by orthopedic surgeons with a reported incidence of 69/100,000 in the United States.^1^ Primary surgical reconstruction results in an ∼9-month intensive recovery postoperatively,^2^ with return to sports allowed after clearance is provided from physicians and physical therapists.^3^ The literature regarding ACL reconstruction with respect to graft type, autograft versus allograft, fixation methods, and revision rates, remains extensive and varied. In a younger patient population, allograft ACL reconstruction is associated with significantly higher failure rates compared with autograft.^4^, ^5^, ^6^

Several different femoral and tibial fixation methods exist for ACL reconstruction with varying literature supporting different techniques. Biomechanical studies suggest grafts with tibial interference fixation provide better time 0 stability when compared to cortical button fixation.^7^ A registry study has also reported lower revision rates with interference fixation on the femoral side coupled with interference screw on the tibial side.^8^ Overall, ACL revision rates range from 7 to 8% at 10 years postoperatively and may be slightly higher in the pediatric population.^9^^,^^10^

To decrease failure risk and enhance protection of the reconstructed ACL, strategies, including ACL graft augmentation with suture or tape, lateral extra-articular tenodesis, and anterolateral ligament reconstruction have been instituted.^11^^,^^12^ The addition of suture or tape to serve as an augment for the ACL graft is easy to perform and does not necessitate a separate open incision. Further, this technique has demonstrated potential in improving both biomechanical^13^ and clinical outcomes following ACL reconstruction.^14^ Tape augmentation increases the strength of the graft complex, demonstrating greater load to failure and reduced elongation.^15^ However, suture or tape augmentation techniques describe incorporating the suture or tape into a separate post from the ACL graft, such as with tibial anchors^16^ or tibial suspensory devices.^17^^,^^18^ These techniques introduce the potential for graft and suture tension mismatch and increase surgical cost due to need for an additional anchor/implant. Here, we describe a postless tape augmentation technique that uses a single tibial fixation implant for both the ACL graft and the tape (Video 1).

## Surgical Technique

**Fig 1:**
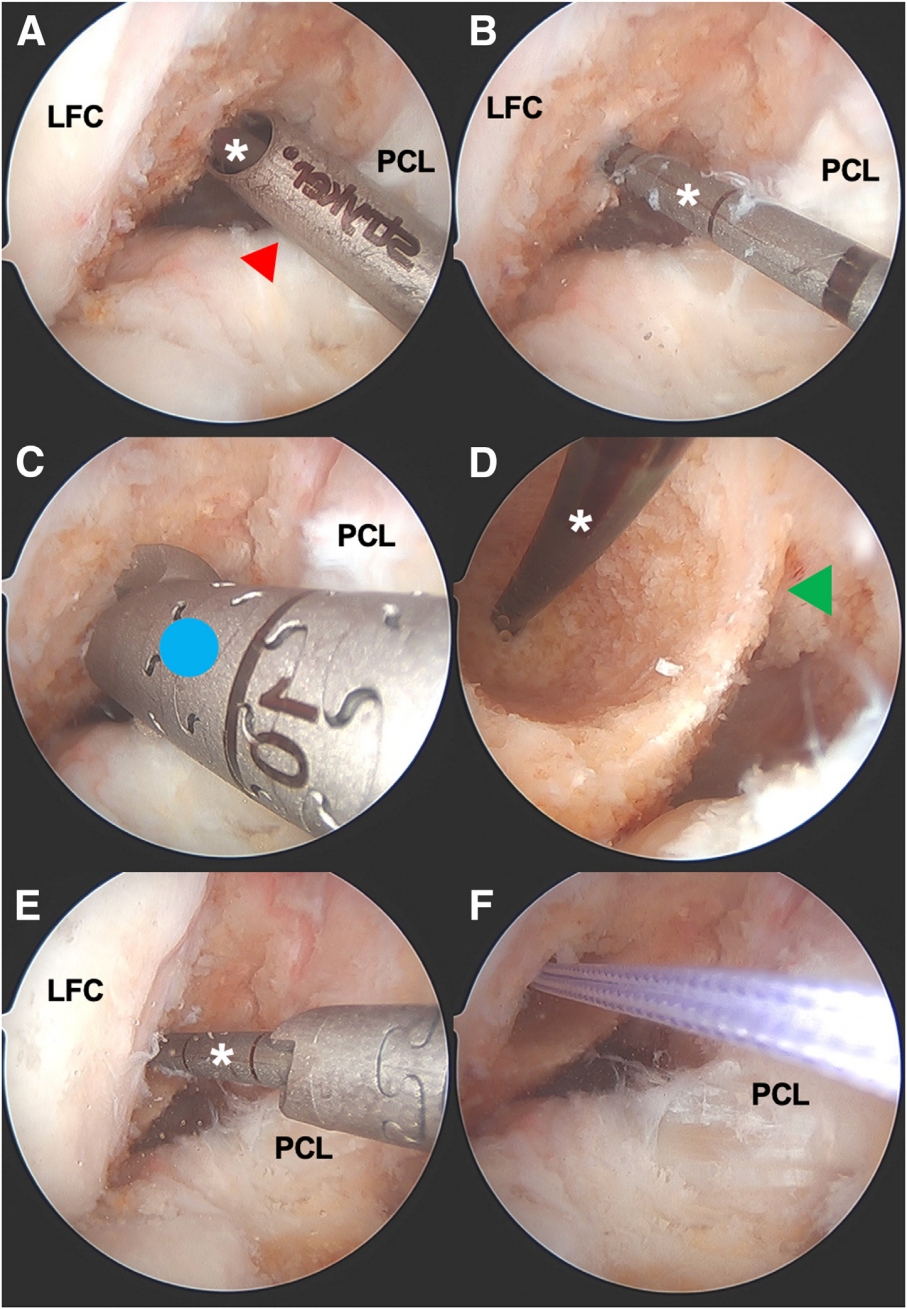
Intraoperative arthroscopic photos of femoral tunnel creation during an anterior cruciate ligament (ACL) reconstruction of the right knee viewing from a standard anterolateral portal with a 30° arthroscope. The femoral tunnel is created via anteromedial portal drilling with flexible curved guides (Stryker VersiTomic; Kalamazoo, MI). (A) The drilling guide (red arrowhead) for flexible drilling is introduced from the anteromedial portal and hooked around the back wall of the lateral femoral condyle. In this case, a 5-mm offset guide is used for a 9-mm graft. The flexible guide pin (white asterisk) is drilled through the guide and through the lateral cortex of the femur and out the skin. (B) The drilling guide is removed and the flexible guide pin (white asterisk) is shown with its location posterior and low in the notch. (C) A flexible cannulated reamer (blue circle) that matches the size of the graft is used over the flexible guide pin. The femoral socket is reamed to 20-25 mm in length, leaving a closed tunnel 8-10 mm from the lateral wall. (D) The arthroscope is placed temporarily through the anteromedial portal to visualize the femoral socket and the back wall (green arrowhead). Here, you can see an intact 1-mm back wall. (E) The arthroscope is brought back to view from the anterolateral portal and a smaller 4.5-mm flexible cannulated reamer is used from the anteromedial portal to ream the lateral femoral cortex to accommodate passage of the femoral cortical button that will be used for suspensory fixation of the graft (Rigidloop; Depuy Synthes, Mitek). (F) A #2 suture is loaded into the eyelet of the flexible guide pin (which is outside the anteromedial portal), and the guide pin is pulled from the lateral aspect of the thigh in order to deliver the suture out of the skin. This will serve as a passing suture for graft passage later. LFC, lateral femoral condyle; PCL, posterior cruciate ligament.

**Fig 2:**
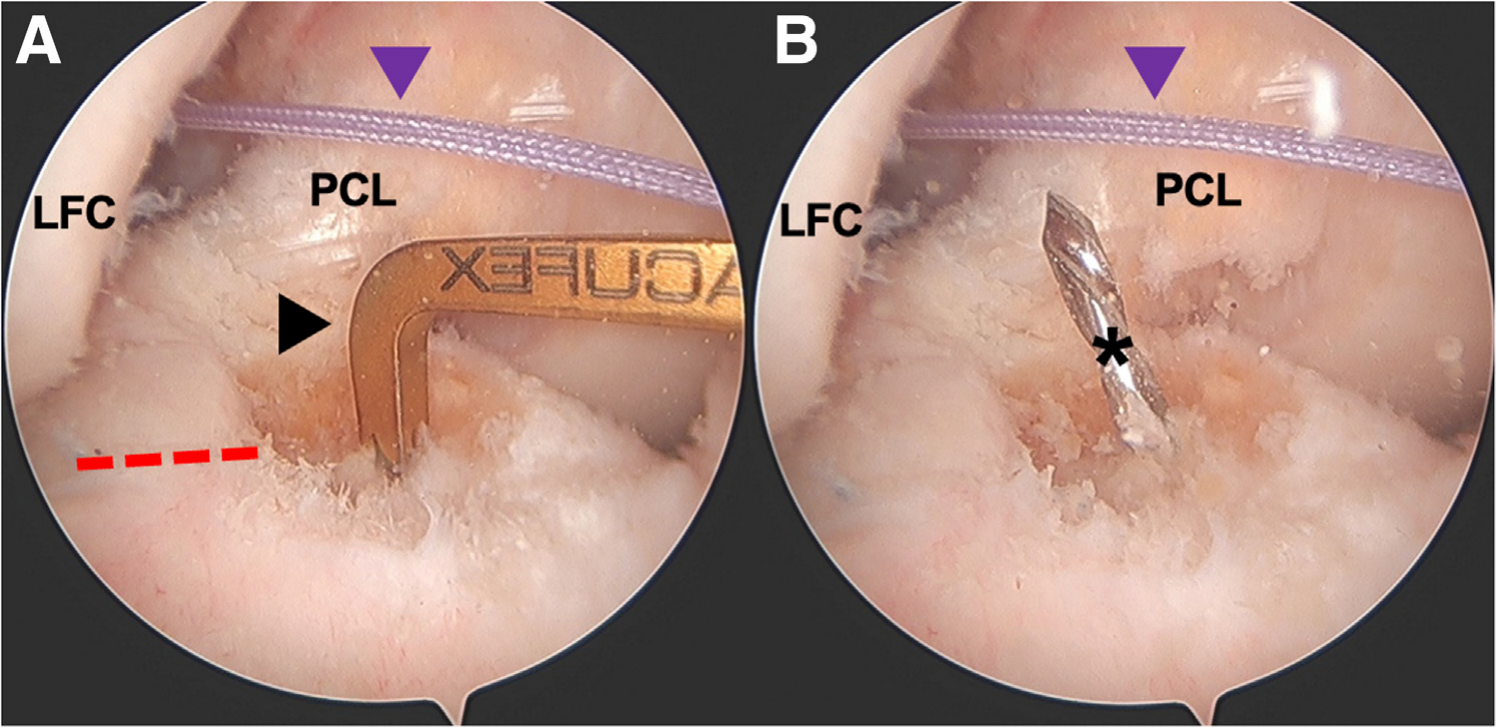
Intraoperative arthroscopic photos of tibial tunnel creation during an anterior cruciate ligament (ACL) reconstruction of the right knee viewing from a standard anterolateral portal with a 30° arthroscope. (A) A tip aiming guide (black arrowhead) is used for tibial tunnel creation. In this case, a tip aiming Acufex guide (Smith & Nephew; London, UK) set to 55° is used. The native ACL tibial footprint, as well as the posterior aspect of the anterior horn lateral meniscus (red dashed line), are used as anatomic landmarks for tunnel creation. (B) The guide pin (black asterisk) is drilled from outside-in through the guide and is visualized. If location is appropriate, a cannulated reamer matching graft size is used over the guide pin to create the tibial tunnel from outside-in. A #2 suture (purple arrowhead) has already been passed through the femoral funnel and is exiting the anteromedial portal and the lateral aspect of the distal thigh. After tibial tunnel creation, this suture is retrieved from the tibial tunnel itself so that it can be used as a passing suture to deliver the graft into the joint. LFC, lateral femoral condyle; PCL, posterior cruciate ligament.

Standard anterolateral (AL) and anteromedial (AM) arthroscopic knee portals are created. Diagnostic arthroscopy begins with a 30° arthroscope from a standard knee anterolateral portal. Any meniscal pathology is first addressed followed by the anterior cruciate reconstruction. Once the ACL remnant stump is debrided, the femoral tunnel is created. Although this technique can be performed with any femoral drilling technique, we prefer to create the femoral tunnel via anteromedial portal drilling with flexible curved guides (Fig 1). Although viewing from the anterolateral (AL) portal with a 30° arthroscope, the flexible anteromedial (AM) guide is introduced from the AM portal and hooked around the back wall of the lateral femoral condyle. For a 9-mm graft, a 5-mm offset AM flexible guide is used. The femoral socket is matched with the graft diameter and typically reamed to 20-25 mm in length, leaving a closed tunnel 8-10 mm from the lateral wall. The tibial tunnel is then created using a tip aiming guide using the posterior aspect of the anterior horn lateral meniscus as a landmark for anatomic tunnel creation (Fig 2). The tibial tunnel is created from outside in and matched to the size of the graft.

**Fig 3:**
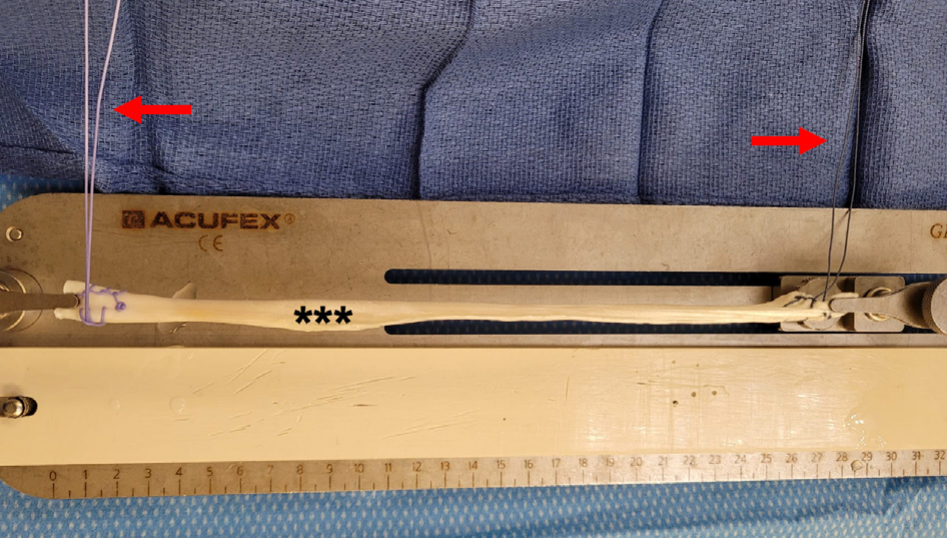
A posterior tibialis tendon allograft (∗∗∗) is sized to a folded diameter of 9 mm. The allograft is placed onto a graft prep station and the two ends of the graft are then each whipstitched with #2 high tensile strength suture (Orthocord; Depuy Synthes, Mitek) (red arrows).

**Fig 4:**
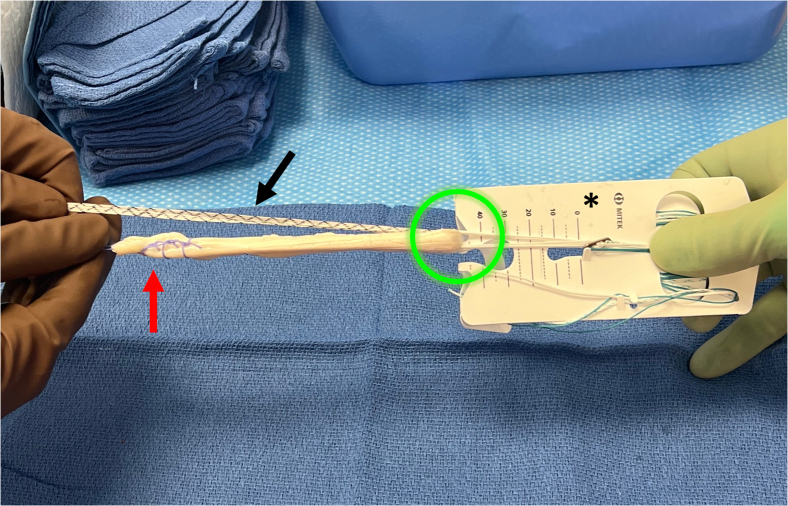
On the back table the surgeon or assistant prepares the allograft. The posterior tibialis tendon allograft is first sized to a folded diameter of 9 mm. The allograft is then whipstitched at both ends with #2 high tensile strength suture (red arrow). The allograft is then loaded onto the femoral button (Rigidloop; Depuy Synthes, Mitek) (∗) by folding the allograft once through the suture loop of the button (green circle). Finally, the 2.5-mm tape suture (DynaTape; Depuy Synthes, Mitek) (black arrow) is folded over at its midpoint through the suture loop of the button in the same fashion as the graft.

This postless tape augmentation technique is performed using suspensory fixation on the femoral side. This technique can be performed with autograft (hamstring, patellar tendon, quadriceps tendon) or allograft (all soft tissue, bone tendon bone, Achilles) using suspensory fixation. In this case example, the decision was made for an all soft-tissue allograft ACL reconstruction using posterior tibialis tendon. The allograft may be prepared by an assistant concurrently, while the surgeon completes the diagnostic arthroscopy and prepares the femoral and tibial tunnels. A posterior tibialis tendon allograft is first sized to a folded diameter of 9 mm. The two ends of the allograft are then each whipstitched with #2 high tensile strength suture (Orthocord; Depuy Synthes, Mitek) (Fig 3). The graft is then loaded onto the femoral cortical button (RIGIDLOOP; Depuy Synthes, Mitek) by folding the graft over once through the suture loop of the button (Fig 4). Next, the 2.5-mm tape suture (DynaTape; Depuy Synthes, Mitek) is simply folded over at its midpoint through the suture loop of the button in the same fashion as the graft.

**Fig 5:**
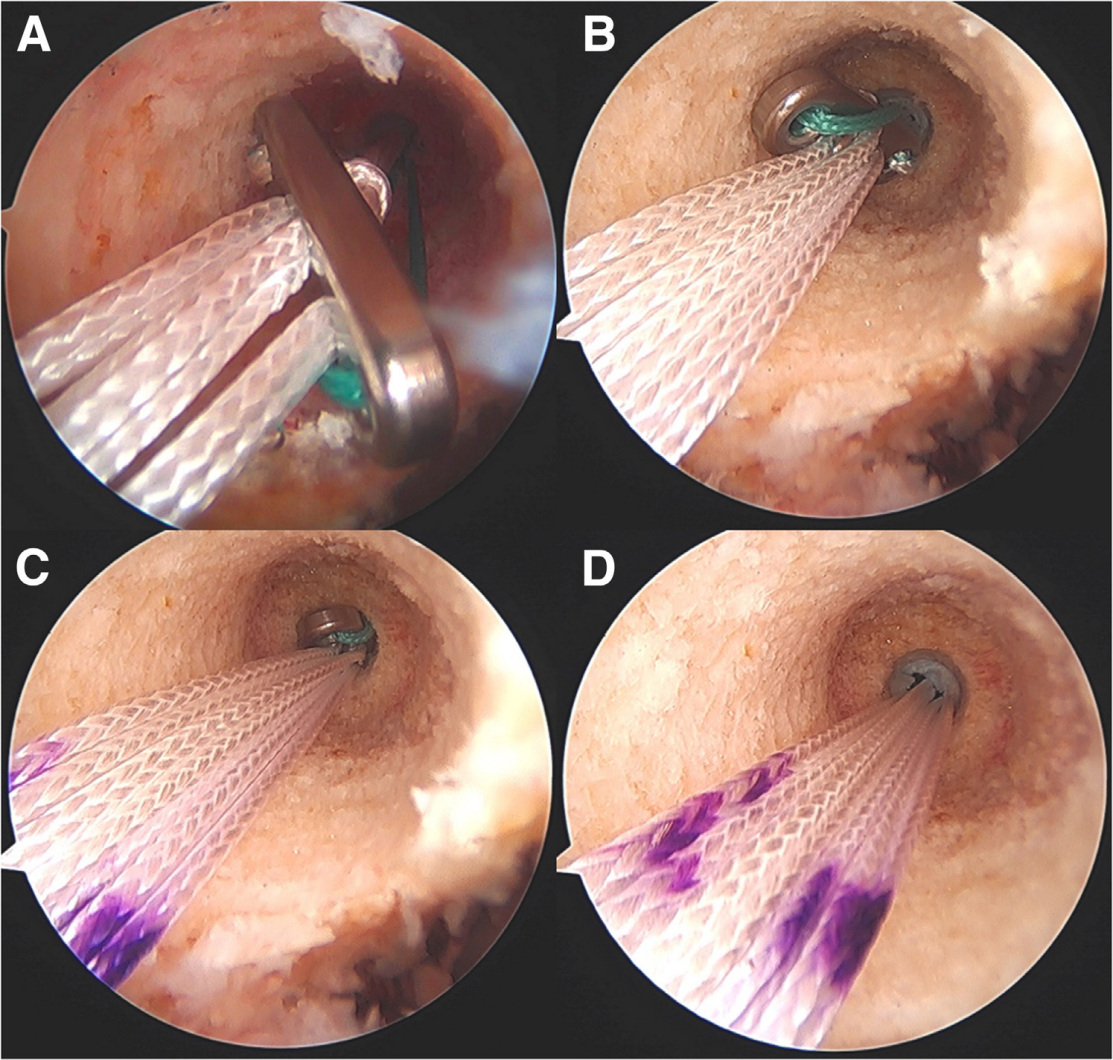
Intraoperative arthroscopic photos during an anterior cruciate ligament (ACL) reconstruction of the right knee viewing from a standard anteromedial portal with a 30° arthroscope showing the progression of the femoral cortical button being passed from the intra-articular space onto the lateral femoral cortex. (A) The femoral cortical button (RIGIDLOOP; Depuy Synthes, Mitek) is in the joint near the femoral socket. (B) The button is advanced retrograde into the femoral socket, which was reamed to the size of the graft. (C) The button is now engaged in the smaller reamed tunnel (4.5 mm), which is just large enough for the button to pass through. (D) The button is now passed through the smaller tunnel and is flipped onto the lateral femoral cortex.

**Fig 6:**
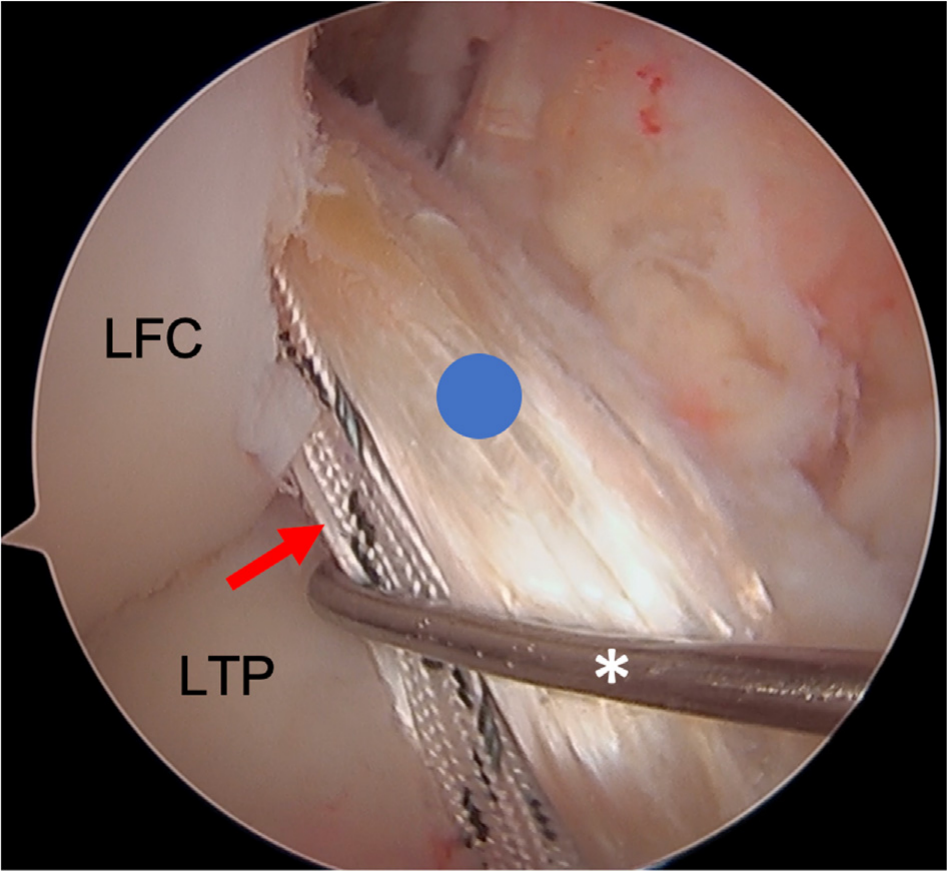
This image shows the view from a standard anterolateral portal with a 30° arthroscope in the patient’s right knee. An arthroscopic probe (∗) is introduced from a standard anteromedial portal and is seen in this image probing the allograft (blue circle) and associated tape augment (red arrow). At this point, the allograft and tape augmentation for the anterior cruciate ligament (ACL) reconstruction is fully docked into the femoral socket. LFC, lateral femoral condyle; LTP, lateral tibial plateau.

After the ACL tunnels have been created, using a passing suture, the femoral button (which is now loaded with the allograft and tape) is then passed into the knee from outside the tibial tunnel, into the joint, and into the femoral tunnel. The button is then flipped onto the lateral femoral cortex (Fig 5). Afterward, the tensioning mechanism of the button is engaged, so that the graft along with the tape is brought into the joint and docked into the femoral socket (Fig 6).

**Fig 7:**
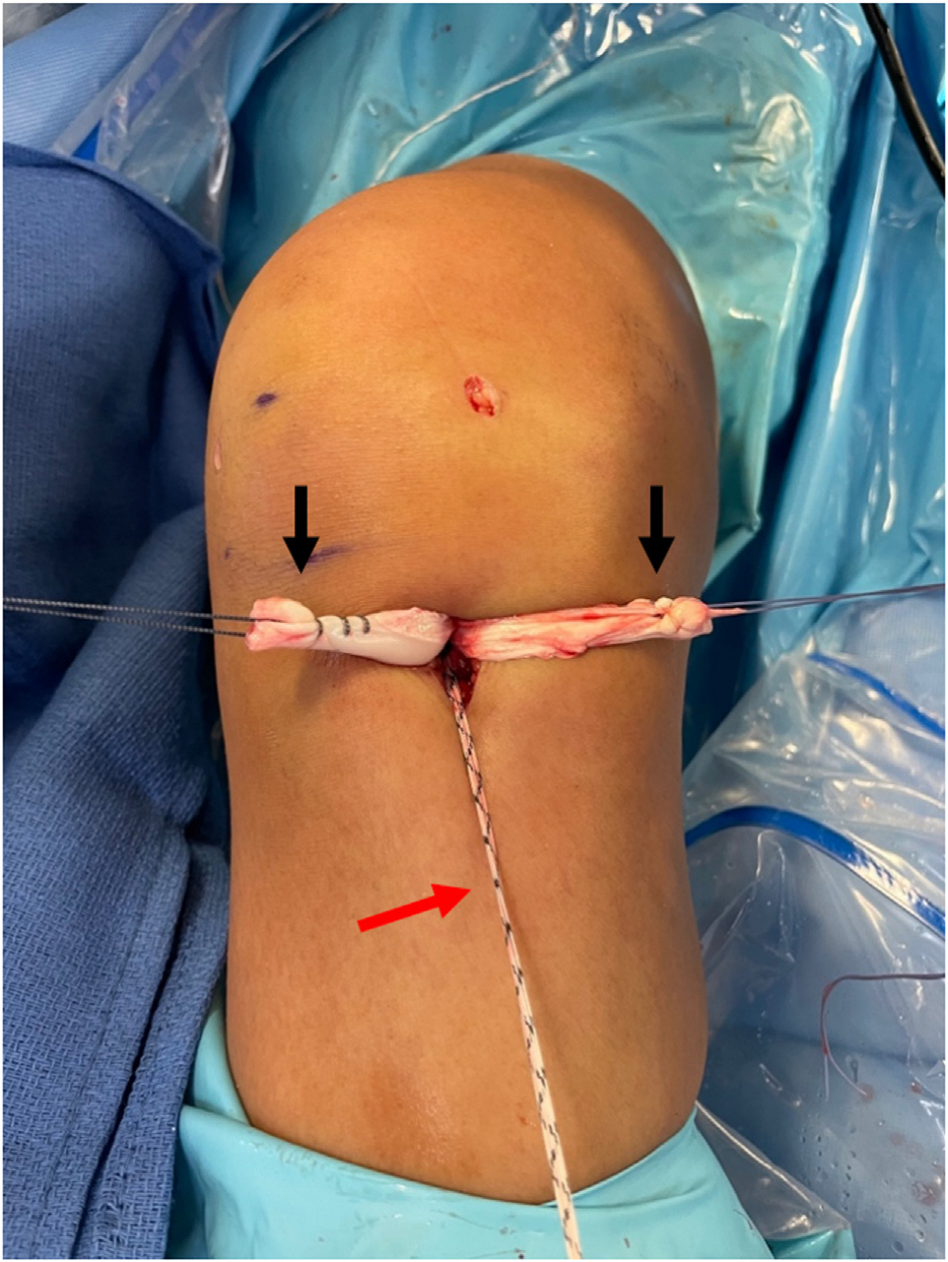
This photo is of the right knee during an anterior cruciate ligament (ACL) reconstruction with postless tape augmentation. The allograft along with the tape has already been secured and docked in the femoral socket using a femoral cortical button (RIGIDLOOP; Depuy Synthes, Mitek). Since the allograft (posterior tibialis tendon) has been folded once over the suture loop of the button, two limbs of the allograft that have previously been whipstitched (black arrows) exit the tibial tunnel along with the two limbs of the tape (red arrow). For this case, the two limbs of the graft are separated, while the two limbs of the tape are kept together to create a three-limb construct where the tibial sheath and screw can be inserted into the center.

**Fig. 8:**
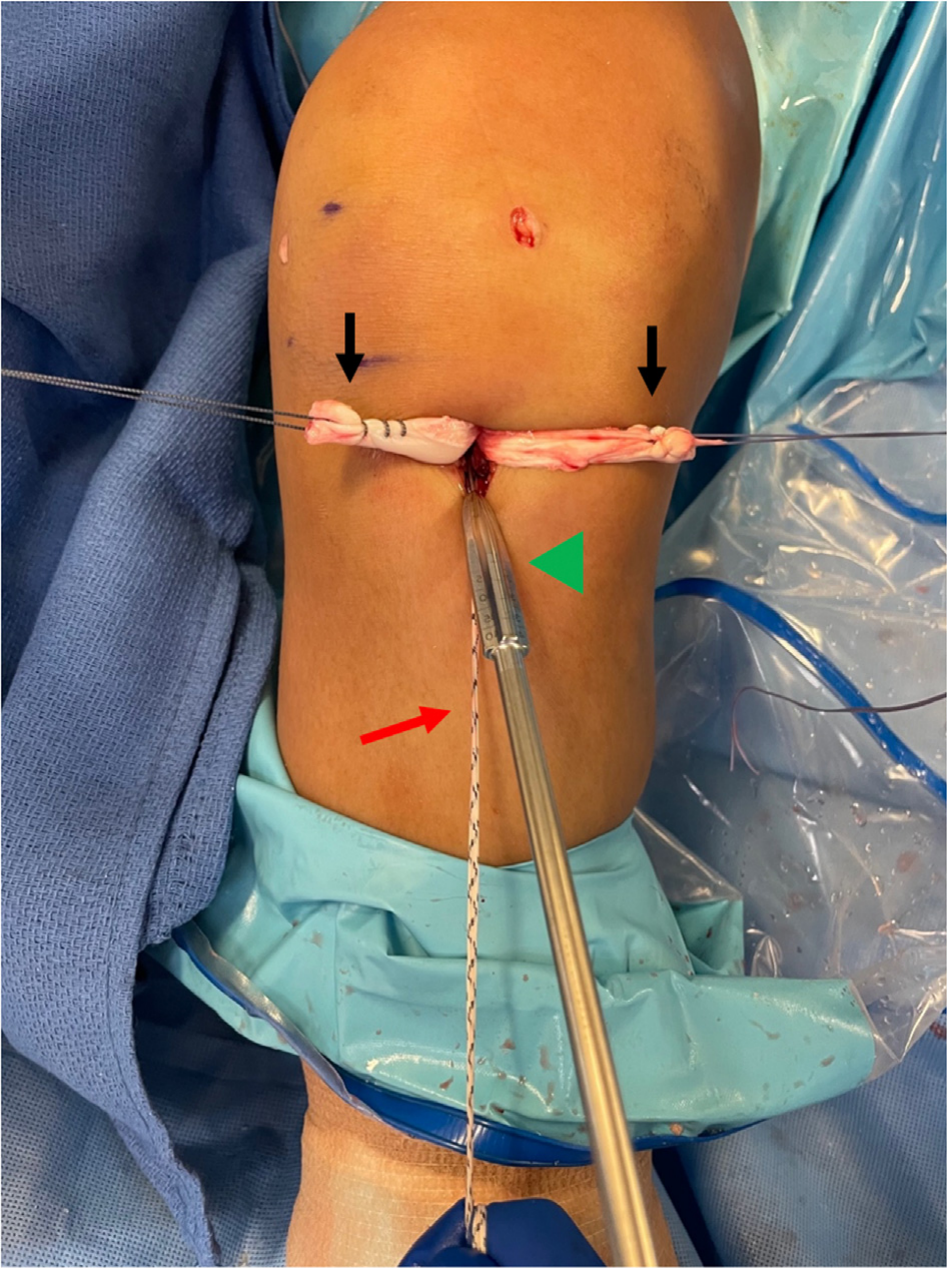
This photo is of the right knee during an anterior cruciate ligament (ACL) reconstruction with postless tape augmentation showing tibial fixation. The surgeon or assistant maintains the knee in 30° of flexion and tensions the two limbs of the allograft (black arrows) and the two limbs of the tape (red arrow). The two limbs of the graft are separated, while the two limbs of the tape are kept together to create a three-limb construct where the tibial sheath and screw (Intrafix Advance; Depuy Synthes, Mitek) can be inserted into the center. First, an appropriately sized dilator is used and advanced into the tibial tunnel to create space between the graft limbs.

**Fig 9:**
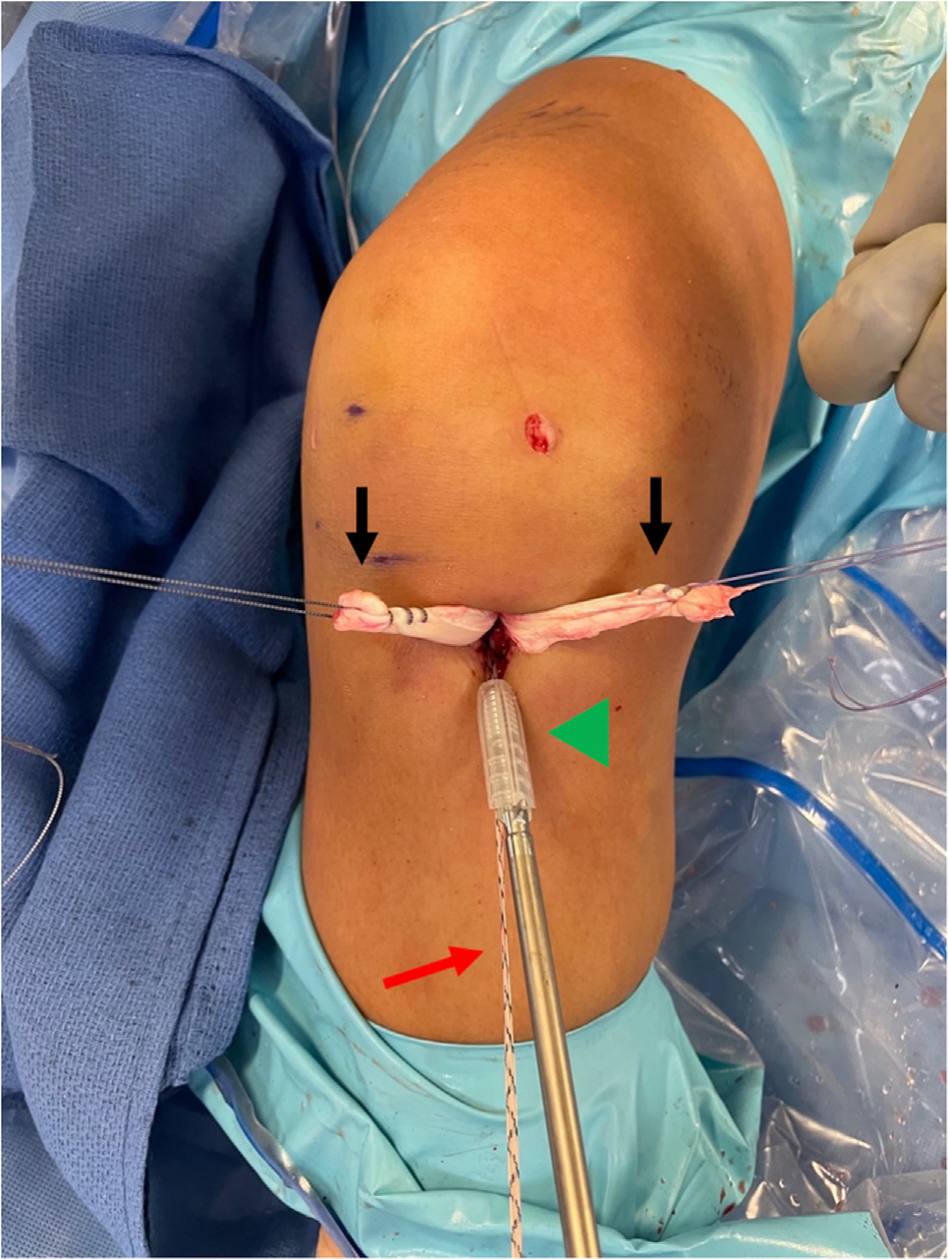
This photo is of the right knee during an anterior cruciate ligament (ACL) reconstruction with postless tape augmentation showing tibial fixation. The tibial sheath and screw (Intrafix Advance; Depuy Synthes, Mitek) are inserted in the center between the two limbs of the allograft (black arrows) and the tape (red arrow). After a dilator is passed, the appropriately sized sheath (green arrowhead) is advanced into the tibial tunnel until it lays flush with the tibial cortex. The sheath and screw will be the same size as the diameter of the tibial tunnel.

**Fig 10:**
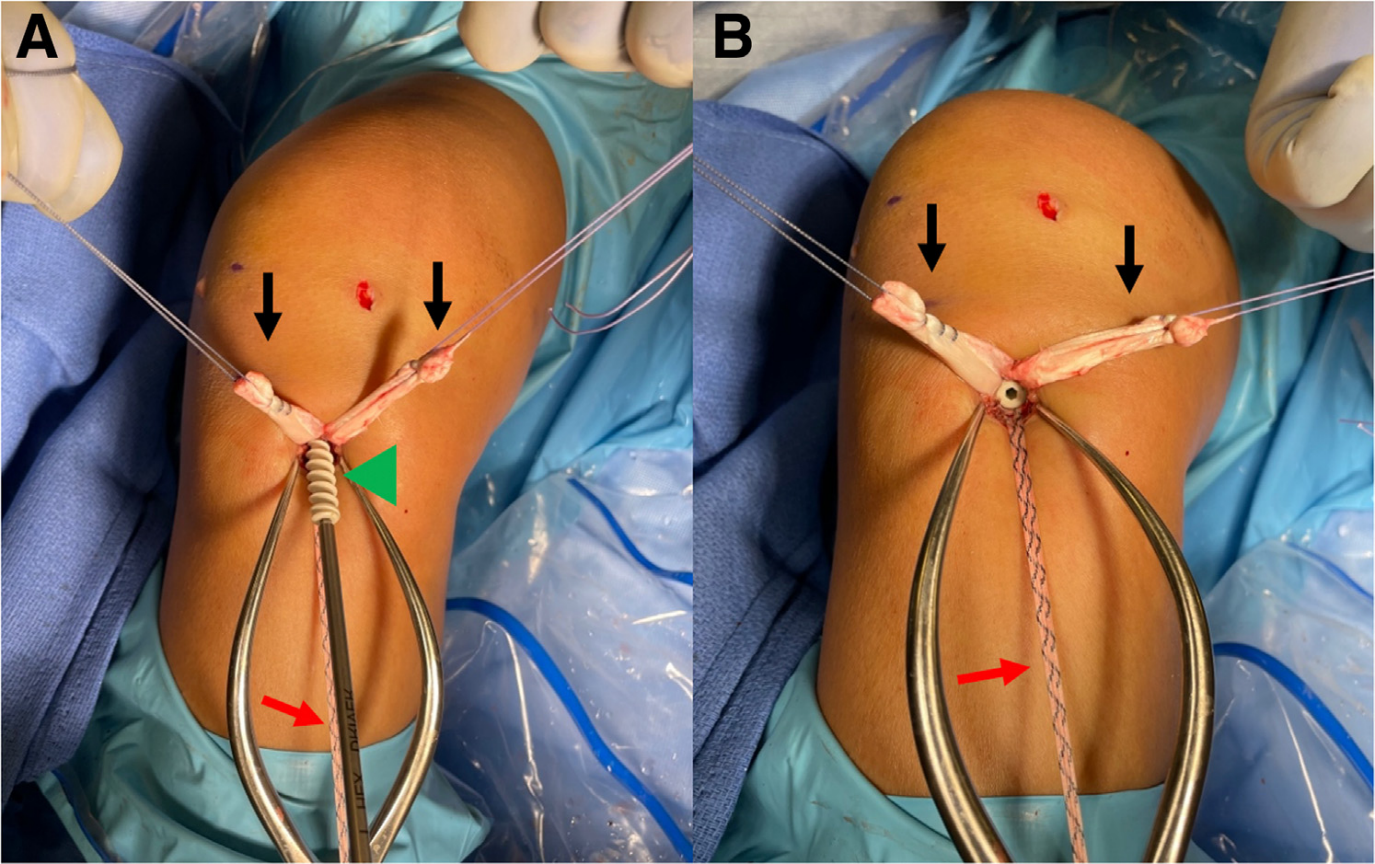
This photo is of the right knee during an anterior cruciate ligament (ACL) reconstruction with postless tape augmentation showing tibial fixation. The tibial sheath and screw (Intrafix Advance; Depuy Synthes, Mitek) is inserted in the center between the two limbs of the allograft (black arrows) and the tape (red arrow). (A) After the sheath is placed, the screw (green arrowhead) is advanced through the sheath. It is essential to maintain tension on allograft and tape during all steps of tibial sheath and screw placement. (B) View of the tibial tunnel aperture after completed fixation showing the sheath and screw lies in between the limbs of the allograft and tape, and the screw and sheath implant is flush with the tibial cortex.

**Fig 11:**
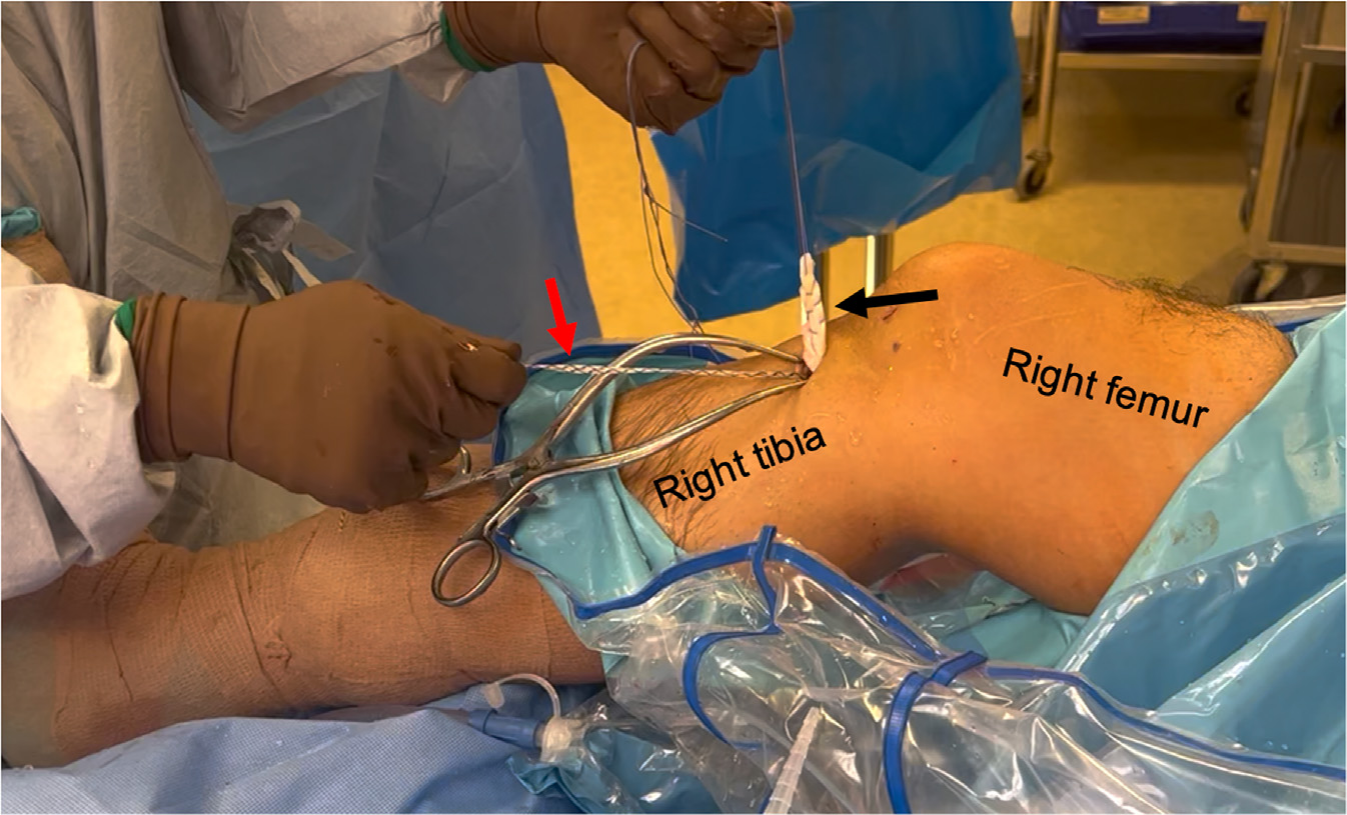
This image is of a patient’s right knee during allograft anterior cruciate ligament (ACL) reconstruction with postless tape augmentation. The allograft (black arrow) along with the tape (red arrow) has been fixed to the tibia with the sheath and screw implant (Intrafix Advance; Depuy Synthes, Mitek). Both the allograft and tape are securely fixed; pulling tension on either the allograft or the tape moves the whole knee. The tape does not slide or creep after fixation.

Once the allograft and tape are docked into the femoral socket, attention is turned toward tibial fixation. A tibial sheath and screw are used for fixation (INTRAFIX Advance; Depuy Synthes, Mitek) of both the graft and augment. We prefer to use polyetheretherketone material for the sheath and screw, but bioabsorbable options are also available. Since the allograft (posterior tibialis tendon) has been folded once over the suture loop of the button, two limbs of the allograft that have previously been whipstitched along with the two limbs of the tape exit the tibial tunnel. We will typically first cycle the graft to remove any creep, and then the surgeon or assistant maintains the knee in 30° of flexion and tensions the two limbs of the allograft as well as the tape. For this case, the two limbs of the graft are separated, while the two limbs of the tape are kept together to create a three-limb construct (Fig 7) where the tibial sheath and screw can be inserted into the center. For other grafts such as an Achilles allograft, the single graft limb can be separated from the two limbs of the tape (which are kept together), and the tibial sheath and screw can be insert in between them. For tibial fixation, first, a dilator is used and advanced into the tibial tunnel to create space between the graft limbs (Fig 8). This is followed by placement of the appropriately sized sheath (typically same size as the tibial tunnel), which is made flush with the tibial cortex (Fig 9). The screw is then advanced into the center of the sheath (Fig 10). It is essential to maintain tension on the graft and tape during all steps of tibial sheath and screw placement. The same surgeon or assistant should be holding the graft and the tape to ensure the same tension is placed on both. Once tibial fixation is complete, the screw and sheath should lie in between the limbs of the allograft and tape and flush with the tibial cortex (Fig 10B). No additional fixation or post is needed, as the sheath and screw construct create a robust interference fixation of both the graft and the tape, which would not be possible with an interference screw alone (Fig 11). Cyclic testing intraoperatively demonstrates no creep of the tape augment in the tibial tunnel. Finally, as the graft and tape are fixed at the same location with the same device, eliminating concern over potential tension mismatch between the graft and tape.

The excess lengths from limbs of the graft and tape are cut flush with the tibial surface. Wounds are irrigated and closed, sterile dressings are applied, a hinged knee brace is placed, and a standard postoperative rehabilitation program for ACL reconstruction is pursued. Advantages and disadvantages (Table 1), as well as pearls and potential pitfalls (Table 2) of this technique are summarized.

**Table 1:** Advantages and Disadvantages of ACL Reconstruction With Postless Tape Augmentation

Advantages	Disadvantages
• Simple to perform and does not alter typical ACL reconstruction technique	• Difficult to fix augment on the femoral side if not using suspensory fixation
• No extra fixation is required for the tape augmentation.	• Tape augmentation is isometric, while ACL reconstruction graft may not be isometric.
• No tension mismatch between the graft and tape	• Potential stress shielding of ACL graft from tape augmentation
• Avoids additional cost of a separate implant or device to be used as a post for augmentation	• Minor increased cost with adding tape suture to the construct

**Table 2:** Pearls and Potential Pitfalls of ACL Reconstruction with Postless Tape Augmentation

Pearls	Potential Pitfalls
• Place the tibial sheath and screw in between the limbs of the graft and the tape (but keeping the limbs of the tape together as one unit). This is to ensure the graft and tape each have interference fit from the sheath implant and bone for secure fixation.	• Failure to equally tension the limbs of the graft in addition to the tape during tibial fixation will lead to tension mismatch.
• Ensure relatively equal tails of the tape limbs after it is passed through the suture loop of the femoral button.	• Burying the tibial screw too deep underneath the tibial cortex can weaken the construct, as cortical fixation is important for the sheath and screw construct.
• The same person (either surgeon or assistant) should tension both the graft and the tape augment simultaneously.	• Performing postless augmentation with a tibial interference screw alone (without a sheath) can result in the tape or graft wrapping around the screw, as well as the screw cutting into the tape or graft.
• Position the tape augment inferiorly in the tibial tunnel and the graft superior in the tunnel with the tibial sheath and screw in the center for better isometry.	

## Discussion

Various surgical modifications have been trialed in the past two decades to minimize ACL graft rerupture, including lateral extra-articular tenodesis,^19^ anterolateral ligament reconstruction,^20^ and “internal brace” with suture or tape augmentation.^21^ Suture or tape augmentation of the reconstructed ACL is a simple procedure that does not require additional open incisions and may serve to protect the naive graft until full incorporation occurs.

Biomechanically, suture or tape augmentation has been shown to significantly increase the force needed to cause graft failure by increasing load sharing between the graft and brace.^22^ The addition of an augment may also increase the diameter of at-risk soft tissue grafts with a diameter of less than 7 mm.^23^ Further, by protecting the native ACL with suture augmentation until incorporation, physical therapy may progress at an accelerated pace in carefully selected patients.^24^ Clinically, augmentation with primary reconstruction has demonstrated consistently strong outcomes in the short and long term.^25^ Schneider et al.^25^ evaluated functional outcomes in 93 patients at 1 year postoperative and found good to excellent functional results with low rate of revision. In a cohort study of 40 patients in each treatment group, Kitchen et al.^14^ demonstrated significantly higher Tegner scores in patients with tape augmentation when compared to those without.

While numerous augmentation techniques have been described in ACL reconstruction literature, all rely on either independent suture anchor tibial placement or individually tensioned suspensory fixation.^16^, ^17^, ^18^ Although these techniques have demonstrated short-term success, relying on independent augment tensioning presents inherent risks. By tensioning the suture or tape augment separately from the graft, a tension mismatch may occur, which may consequently overconstrain or underconstrain the knee. This can also cause stress shielding to the ACL graft and inhibit graft incorporation. Furthermore, the addition of another implant such as a suture anchor to be used as a post for the augment will add cost to the surgical procedure. For surgeons operating in cost-conscious surgery centers, this can be a significant deterrent for augmentation.

In this technical note, we present a technique that benefits from the protection of tape augmentation while eliminating the potential of tension mismatch between the graft and augment. By using a postless interference fixation with a sheath and screw at the tibial tunnel, the graft and tape can be equally tensioned, avoiding the risks of augment over- or under-constraint. Intraoperative testing of the strength of fixation confirms the integrity of this system as no creep of the tape was found during stress testing. Avoidance of individual or independent graft and augment fixation may provide optimal protection of the young graft prior to incorporation, but more longitudinal research is required to investigate ACL reconstruction augmentation techniques.

## Supplementary Data

ICMJE author disclosure forms

Video 1 This technique shows the case of an allograft anterior cruciate ligament (ACL) in a right knee with postless tape augmentation. The two ends of a posterior tibialis tendon allograft are each whipstitched with #2 high tensile strength suture (Orthocord; Depuy Synthes, Mitek). The graft is then loaded onto the femoral cortical button (RIGIDLOOP; Depuy Synthes, Mitek) by folding the graft over once through the suture loop of the button. A 2.5-mm tape suture (DynaTape; Depuy Synthes, Mitek) is also loaded through the suture loop of the button. Standard anterolateral (AL) and anteromedial (AM) arthroscopic knee portals are created. Diagnostic arthroscopy begins with a 30° arthroscope from a standard knee anterolateral portal. After a femoral socket and a tibial tunnel are created, the femoral button (which is now loaded with the allograft and tape) is then passed into the knee from outside the tibial tunnel, into the joint, and into the femoral tunnel. The femoral button is flipped onto the lateral femoral cortex, the graft along with tape is advanced and docked into the femoral socket using the tensioning mechanism of the button. A sheath and screw (Intrafix Advance; Depuy Synthes, Mitek) are used for tibial fixation. The surgeon or assistant maintains the knee in 30° of flexion and tensions the two limbs of the allograft, as well as the tape simultaneously, so that the sheath and screw can be inserted in the center between the limbs. First, a dilator is used and advanced into the tibial tunnel, followed by the placement of the appropriately sized sheath. The screw is then advanced through the sheath. It is essential to maintain tension on allograft and tape during all steps of tibial sheath and screw placement. No additional fixation is required as both the graft and the tape are well fixed with the sheath and screw. A clamp placed on the tape at the tibial tunnel aperture shows no creep during range of motion. The graft and tape are fixed at the same location with the same device eliminating concern over potential tension mismatch between the graft and tape.
